# An in vitro platform for quantifying cell cycle phase lengths in primary human intestinal epithelial cells

**DOI:** 10.1038/s41598-024-66042-9

**Published:** 2024-07-02

**Authors:** Michael J. Cotton, Pablo Ariel, Kaiwen Chen, Vanessa A. Walcott, Michelle Dixit, Keith A. Breau, Caroline M. Hinesley, Katarzyna M. Kedziora, Cynthia Y. Tang, Anna Zheng, Scott T. Magness, Joseph Burclaff

**Affiliations:** 1 Joint Department of Biomedical Engineering, University of North Carolina at Chapel Hill and North Carolina State University, Chapel Hill, NC 27599 USA; 2 Microscopy Services Laboratory, Department of Pathology and Laboratory Medicine, University of North Carolina at Chapel Hill, Chapel Hill, NC 27599 USA; 3 Department of Cell Biology and Physiology, University of North Carolina at Chapel Hill, Chapel Hill, NC 27599 USA; 4 Center for Gastrointestinal Biology and Disease, University of North Carolina at Chapel Hill, Chapel Hill, NC 27599 USA; 5 Department of Cell Biology, Center for Biologic Imaging (CBI), University of Pittsburgh, Pittsburgh, PA 15260 USA; 6 School of Medicine, University of North Carolina at Chapel Hill, Chapel Hill, NC 27599 USA

**Keywords:** Cell cycle phase, Intestinal stem cell, Fluorescent reporter, Live imaging analysis, Collagen press, Biological techniques, Cell biology, Stem cells, Gastroenterology

## Abstract

The intestinal epithelium dynamically controls cell cycle, yet no experimental platform exists for directly analyzing cell cycle phases in non-immortalized human intestinal epithelial cells (IECs). Here, we present two reporters and a complete platform for analyzing cell cycle phases in live primary human IECs. We interrogate the transcriptional identity of IECs grown on soft collagen, develop two fluorescent cell cycle reporter IEC lines, design and 3D print a collagen press to make chamber slides for optimal imaging while supporting primary human IEC growth, live image cell cycle dynamics, then assemble a computational pipeline building upon free-to-use programs for semi-automated analysis of cell cycle phases. The PIP-FUCCI construct allows for assigning cell cycle phase from a single image of living cells, and our PIP-H2A construct allows for semi-automated direct quantification of cell cycle phase lengths using our publicly available computational pipeline. Treating PIP-FUCCI IECs with oligomycin demonstrates that inhibiting mitochondrial respiration lengthens G1 phase, and PIP-H2A cells allow us to measure that oligomycin differentially lengthens S and G2/M phases across heterogeneous IECs. These platforms provide opportunities for future studies on pharmaceutical effects on the intestinal epithelium, cell cycle regulation, and more.

## Introduction

Intestinal epithelial cells (IECs) exhibit dynamic cell cycle control. Intestinal stem cells (ISCs) and rapidly dividing transit amplifying (TA) cells proliferate at different rates at homeostasis, with predicted average doubling times of 24 h and 12 h, respectively^1^. Exogenous nutrients and dietary molecules alter cell cycle^2^, and cells cycle differently after acute or chronic injuries or disease^3,4^. The importance of individual cell cycle phases is becoming increasingly evident—for example, stem cells are shown to make fate decisions in response to stimuli received in G1 phase^5–7^. Studying the effects of changes to cell cycle phases and their regulatory mechanisms are active fields, yet no platforms currently exist for visualizing or tracking cell cycle phase lengths in live human IECs.

Multiple techniques have been used to discern cell cycle lengths in IECs, with nearly all data arising from model organisms or immortalized cell lines. Early studies tracked proliferative lineages by counting mitotic indices or pulsing with tritiated thymidine, with studies from the 1970s using percent labelled mitosis (PLM) or vincristine mitotic accumulation (VCR) experiments in mice and rats to estimate that small intestinal crypt cells cycle every 10–24 h depending on position^8^. Schepers et al.^9^ paired EdU pulsing with PH3 immunostaining to show that mouse ISCs divide every 21.5 h on average. These methods all estimate the full cell cycle, not individual phase lengths. Similarly, studies on cell cycle perturbation often rely on staining for proteins such as Ki67 or PCNA or pulsing cells with nucleotide analogs (BrdU or EdU) which mark cells synthesizing DNA during the pulse. While these strategies are useful for comparing overall proliferation between populations, they lack information about individual cell cycle phase lengths.

A major method for analyzing cell cycle phases uses flow cytometry to analyze DNA content in cells. The proportion of cells in each phase at a given time can be calculated by counting cells with 2n DNA (G1/G0 phase), 4n DNA (G2/M phase), or intermediate levels (S phase). However, this method does not measure cell cycle phase lengths and can miss valuable information. For example, ratiometric techniques would be unable to determine if a perturbation shortens or lengthens all phases of the cell cycle—instead they only inform if one phase is changed more than the others. For more nuanced results, and to compare cell cycle lengths across individual cells, directly measuring cell cycle phase lengths is optimal.

An improved strategy for directly measuring cell cycle phase lengths arose in 2008 with the FUCCI cell cycle reporter gene^10^. This construct caused cells to express different fluorescent reporters in G1 and S/G2/M phases. Several iterations followed, making phase change readouts more concise and visualizing G1, S, and G2/M phases separately^11–13^. These genes have been used in the intestine, largely utilizing mice engineered to ubiquitously express the reporter^14^. Researchers have used these tools to demonstrate that murine ISCs reside largely within the G1 phase^15^ and to measure ratios of cells within each cell cycle phase at individual timepoints, replicating DNA analysis assays but in live cells^16^. One study used murine FUCCI2 cells to trace individual cells to track cell cycle lengths with regards to circadian rhythm^17^. We expanded on this model to engineer a platform to track primary human IECs in an easy-to-image monolayer platform.

This study uses our primary human IEC monolayer culture system^18,19^ with two engineered cell cycle reporters to directly measure cell cycle phases in freely cycling IECs by snapshot analysis of single images or by live imaging to directly quantify phase lengths. We interrogated the transcriptomic identity of IECs cultured on soft collagen, developed a collagen molding process to optimize chamber slides for confocal microscopy of primary IECs, generated two genetically engineered primary human IEC cell cycle reporter lines, and compiled a computational analysis pipeline building on publicly available software. Importantly, by using only two fluorescent channels and publicly available analysis software, our platform maintains high adaptability for diverse projects and provides an easy-to-implement and open-source analysis pipeline.

## Results

### Cultured human IECs maintain markers of proliferation and stemness

**Figure 1:**
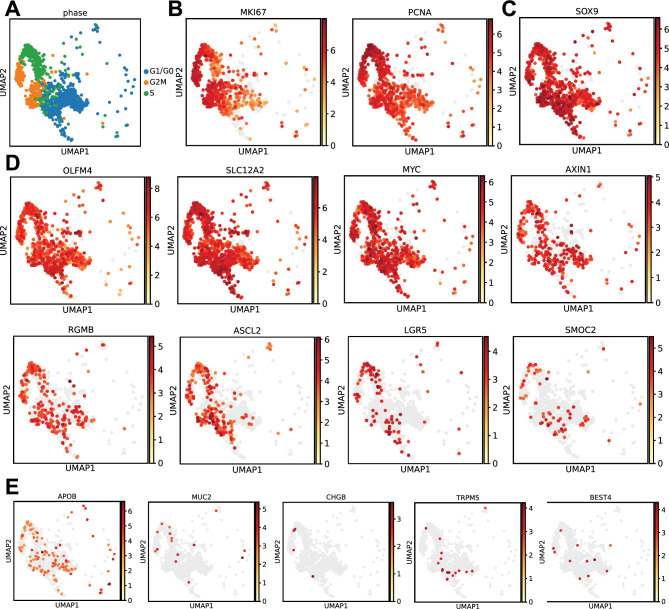
Primary monolayer transcriptomics. (**A**) UMAP showing computationally defined cell cycle phase across single cells dissociated from intestinal monolayers grown on soft collagen in N = 1 well. (**B**) UMAPs for the same cells as in (**A**) showing gene expression (darker red = higher expression per cell, light gray = no detectable signal) for cell cycle genes *MKI67* and *PCNA*; (**C**) General stem/progenitor marker *SOX9*; (**D**) Intestinal stem cell genes *OLFM4, SLC12A2, MYC, AXIN1, RGMB, ASCL2, LRG5, SMOC2;* and (**E**) Differentiated cell marker genes *APOB*, *MUC2*, *CHGB*, *TRPM5*, *BEST4*.

We previously showed how human ISCs plated on soft collagen matrices form self-replicating monolayers that can be passaged numerous times^18,19^ and genetically engineered to express fluorescent reporter genes^20,21^. However, the transcriptomic identity of these undifferentiated monolayers has not been demonstrated. To characterize the monolayers at the single cell level, we analyzed the single cell transcriptome of IECs that were grown on a 1 mm-thick patty of soft collagen to 70% confluence to maintain proliferative populations^22^. According to their transcriptomic signatures, 54.2% of IECs were found to reside in G2/M or S phases (Fig. 1A), demonstrating their proliferative state. The majority of all IECs were defined as actively cycling via detectable expression of *MKI67* and *PCNA* in 63.8% and 74.6% of the cells, respectively (Fig. 1B). Nearly all IECs (81.2%) expressed the stem/progenitor marker *SOX9* (Fig. 1C), and genes from the human ISC transcriptional signature^23^ were expressed across the IECs, with *OLFM4* (81.5%), *SLC12A2* (94.5%), and *MYC* (70%) present in the majority of IECs and *AXIN1* (28.9%), *RGMB* (23.1%), *ASCL2* (22.5%), *LGR5* (9.1%), and *SMOC2* (6.6%) present in more constrained populations (Fig. 1D). Markers of differentiated cells were only detected in small subsets of IECs (absorptive enterocytes, *APOB* in 12.3%; goblet cells, *MUC2* in 1.5%; enteroendocrine cells, *CHGB* in 0.4%; Tuft cells, *TRPM5* in 1.8%; BEST4^+^ cells, *BEST4* in 1.1%; and Paneth cells, *DEFA5*—no expression) (Fig. 1E), confirming the undifferentiated nature of these cultured IECs. These findings indicate that IEC monolayers are predominantly comprised of > 80% stem and progenitor cells with sporadic cells of differentiated lineages.

### An engineered press prevents the collagen meniscus for optimal imaging

**Figure 2:**
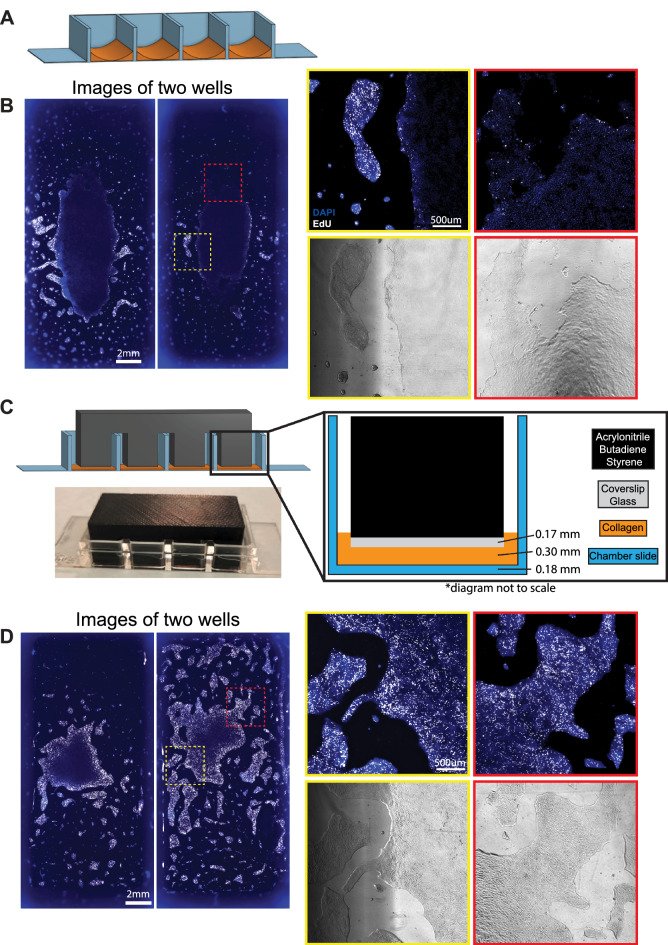
Designing the collagen press. (**A**) Model of meniscus formation following freely poured collagen (orange) setting in chamber slides. (**B**) Immunofluorescence of nuclei (DAPI, blue) and proliferation (EdU uptake, white) in two separate wells of IECs in chamber slides poured with no press. Insets show differences in proliferative capacity and cellular spacing across areas of the same chamber well after 3 days of culture. Brightfield images of the insets show cell coverage (shadows result from chamber slide walls, not collagen or cellular differences). (**C**) Model for 3D-printed collagen press designed to sit across chamber slide walls and reach into wells to prevent meniscus formation. Cover slip glass glued to the leg bottoms forms a smooth surface. Photograph of a collagen press on a chamber slide. (**D**) Immunofluorescence of nuclei (DAPI, blue) and proliferation (EdU uptake, white) in IECs grown in two separate chamber slides using the 3D-printed collagen press. Insets indicate uniform proliferation across colonies.

Collagen hydrogel can be used to create a soft culture substrate (~ 9 kPa) necessary to preserve ISC self-renewal and reduce differentiation^18^. To minimize the distance to the objective (maximizing imaging signal and focus) and allow broad use across confocal microscopes without long working objectives, decreasing amounts of collagen were applied to Ibidi µ-Slide 4 Well chamber slides with a #1.5 polymer coverslip base (170 µm thick). The collagen created a meniscus, which presented a challenge to accurately image across large areas of a non-planar surface caused by the meniscus. The curvature impaired imaging at the edges as the z-plane had a significant slope. Moreover, the center of the meniscus produced a very thin collagen layer with lower volumes of collagen that was insufficient to shield ISCs from the high stiffness of the polystyrene slide, resulting in reduced ISC proliferation capacity (Fig. 2A). This caused stark phenotypic differences across the well, with nuclei spaced further apart and most cells on the central thin collagen exiting the cell cycle, as seen by the decreased EdU uptake even at the edges of the monolayer (Fig. 2B). By contrast, the meniscus edges had thick collagen which supported IEC proliferation, but the cells were on a curved surface and upper regions exceeded the focal range. The different proliferative and cell density phenotypes across the meniscus indicated that the prototype system caused heterogenous IEC growth, substantially decreasing reproducibility within experiments.

To create a flat collagen substrate sufficiently thin to enable live confocal imaging while supporting IEC self-renewal, we developed a collagen press to hold the surface of the collagen flat while it solidified (Fig. 2C). The press was 3D printed (Supplemental File 1), then basal surfaces that contact the collagen were capped with coverslip-thickness glass to create a flat collagen surface and reduce adhesion to the collagen for easy removal (Fig. 2C). The press was designed to rest on the walls separating the slide chambers to create a 0.3 mm gap for the collagen to fill and solidify. IECs cultured on the resulting flat collagen grew more evenly, with uniform thick bands of proliferating cells along the edges of all colonies, even those at the chamber center (Fig. 2D). Avoiding the meniscus also allowed less cells to gather at the center, resulting in more small, isolated colonies that are optimal for live imaging as they minimize contact inhibition during the imaging timeframe. This platform allows for reproducible and consistent maintenance of primary human IECs in four wells to allow for multiple concurrent perturbations, with a minimal working distance to allow for use across confocal microscopes.

### PIP-FUCCI reporter IECs define cell cycle phase from single images

**Figure 3:**
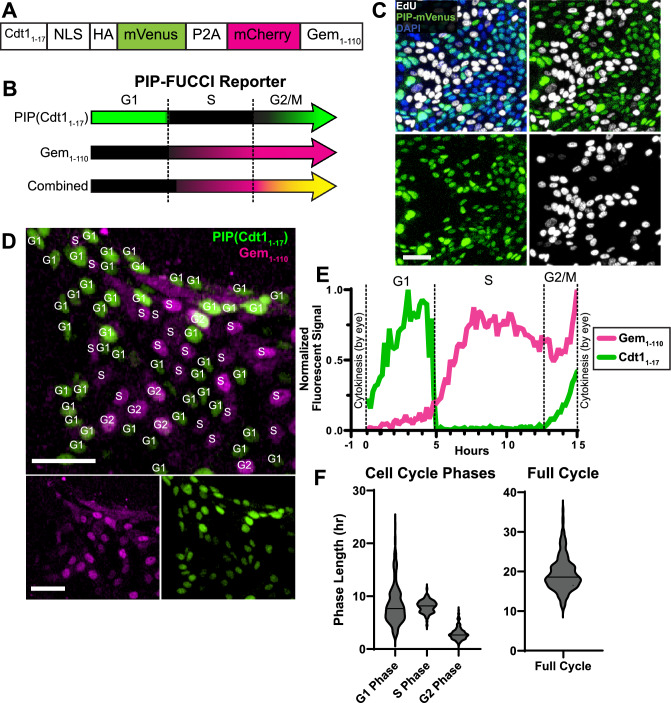
PIP-FUCCI construct and analysis. (**A**) Schematic of PIP-FUCCI genetic construct, (**B**) Schematic of dynamic reporter colors throughout the cell cycle in PIP-FUCCI cells. (**C**) PIP-H2A cells stained for EdU following 1 h EdU pulse. Boxes show individual colors (DAPI, blue; EdU, white; PIP-mVenus, green). (**D**) Representative image of growing PIP-FUCCI human IEC monolayers. Scale bar = 50 µm. Cell cycle phase for each nucleus is superimposed, visually defined by mCherry and mVenus signal. (**E**) Example fluorescence data from an individual PIP-FUCCI nucleus over its full cell cycle. (**F**) Violin plots denoting cell cycle phase lengths across 196 IECs from N = 2 wells using the PIP-FUCCI reporter.

To visualize cell cycle phases in live human IECs, we adapted the recently published PIP-FUCCI fluorescent cell cycle reporter, which has been shown to delineate cell cycle phase changes with high accuracy^11^. The PIP-FUCCI construct consists of two reporters for identifying cell cycle phases. The Cdt1_1-17_-mVenus fusion has strong fluorescence in G1, rapid degradation (< 30 min) to start S Phase, then increasing fluorescence in G2 phase. The Gem_1-110_-mCherry fusion has negligible fluorescence in G1 then fluoresces in the S and G2/M phases through cytokinesis, at which point it rapidly dims (Fig. 3A,B). We engineered the PIP-FUCCI construct onto a PiggyBac transposase plasmid, transfected this plasmid into human IECs, and selected stable clones using our optimized protocols for primary human IEC monolayers^20^. To validate that the PIP-mVenus reporter faithfully marks G1 and G2/M phases but not S phase, clonal cells received a one-hour EdU pulse to mark cells in S phase. Fluorescence microscopy confirmed that EdU^+^ cells were distinct from mVenus-positive cells (Fig. 3C), confirming that the construct was accurately reporting phase changes. To quantify cell cycle phase lengths in growing cells, PIP-FUCCI IECs were thinly seeded onto collagen hydrogels then imaged starting 24 h post-plating to mitigate potential contact inhibition. Since the PIP-FUCCI construct fluoresces distinct color combinations in each cell cycle phase, phases for all cells can be proscribed from a single snapshot by defining whether nuclei have coloration in each channel above background (Fig. 3D).

To determine if this platform could be used to directly measure cell cycle phase lengths in freely cycling human IECs, we live imaged cells for 48 h by confocal microscopy. Fluorescence profiles of individual nuclei were tracked across each 10 min frame interval, with mVenus and mCherry levels used to define cell cycle phases (Fig. 3E). However, a gap exists in early S Phase where both reporters are very dim across 1–3 frames^11^. With no other nucleus marker and given the tightly packed and dynamically moving nature of primary human IEC monolayers, we were unable to automatically track nuclei through the full cell cycle using publicly available programs. Instead, nuclei were tracked by hand through all frames for a full cell cycle to observe reporter changes and determine phase lengths (Fig. 2E). Our analysis of 196 IECs showed that freely cycling PIP-FUCCI IECs had a median total cell cycle length of 18.6 h, with first and third quartile lengths of [16.5, 21.5]. Median lengths for individual cell cycle phases were 7.7 h [5.8, 10.3] for G1 phase, 8.2 h [7.2, 8.8] for S phase, and 2.6 h [2.2, 3.3] for G2/M phases (Fig. 3F). Sample files for analyzing cell cycle phases using PIP-FUCCI IECs are available as Supplementary Files 2–4. While PIP-FUCCI IECs can be used to quantify cell cycle phase lengths from live imaging, the analysis is low throughput and requires significant manual input. Instead, this reporter is optimal for defining cell cycle phases of human IECs using single snapshot images of living or fixed cells.

### PIP-H2A human IEC reporters allow for automated cell tracking to measure cell cycle phases

**Figure 4:**
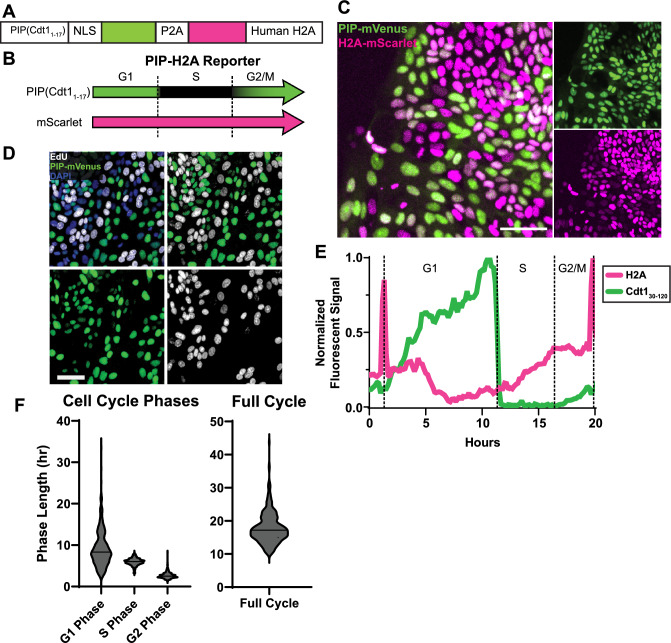
PIP-H2A construct and imaging. (**A**) Schematic of the PIP-H2A genetic construct, (**B**) Schematic of dynamic reporter colors throughout the cell cycle in PIP-H2A cells. (**C**) Representative fluorescence image of the PIP-H2A reporter in monolayers. Scale bar = 50 µm. (**D**) PIP-H2A cells stained for EdU following 1 h pulse. Boxes show individual colors (DAPI, blue; EdU, white; PIP-mVenus, green). (**E**) Example fluorescence data from an individual PIP-H2A nucleus over its full cell cycle. (**F**) Violin plots denoting cell cycle phase lengths across 284 IECs from N = 2 wells using the PIP-H2A reporter.

We next designed a reporter construct to allow for automated analysis of cell cycle phase lengths in live human IEC monolayers using only two fluorescent channels. Our construct labeled all nuclei with a constitutive fluorescent reporter gene for tracking cells alongside the same PIP-mVenus reporter gene used in the PIP-FUCCI construct to precisely define onset of S and G2 phases. For a bright nuclear reporter, we combined the mScarlet-tagged human histone H2A gene^24^ with the original PIP (Cdt1_1–17_-mVenus fusion), creating a novel PIP-H2A reporter line (Fig. 4A). Importantly, this construct is not designed for identifying cell cycle phases from single snapshots, as both G1 and G2 phases exhibit the same fluorescence signature (Fig. 4B). Rather, the PIP-H2A reporter was specifically designed for quantifying cell cycle phase lengths from live imaged cells. Constitutive H2A-mScarlet signal provided the ability to constantly track nuclei and the PIP-mVenus signal enabled precise identification of G1 (high mVenus), S (rapid loss of mVenus), and G2/M phases (increasing mVenus). With constant H2A-Scarlet expression, cytokinesis can easily be observed to define the beginning and end of full cell cycles.

Clonal PIP-H2A IECs all showed mScarlet fluorescence in a snapshot, with mVenus fluorescence lacking in a subset of cells, as expected (Fig. 4C). Interestingly, we noted variable mScarlet brightness that appeared inverse to mVenus signal (Fig. 4C,E). To validate PIP-mVenus reporter function, cells were treated with a one-hour EdU pulse to mark cells in S phase. Fluorescence microscopy confirmed that cells marked by EdU were distinct from cells expressing mVenus (Fig. 4D), validating that the construct was accurately reporting the beginning and end of S phase.

We developed an automated image analysis pipeline to increase throughput of analyzing cell cycle lengths in these IECs. The approach was designed to computationally identify nuclei from live imaging results, track each nucleus across timepoints, then analyze the mVenus and mScarlet signal changes in each nucleus over time. For cells tracked over a full cell cycle, fluorescent color changes can be aligned to cell cycle phase transitions. To quantify cell cycle phase lengths in freely cycling primary human IECs, cells were plated sparsely on pressed collagen chamber slides, cultured for two days, then live imaged for 48 h with imaging at 10-min intervals. To optimize automatic tracking of nuclei across timeframes, we added the signal intensities from the mScarlet and mVenus channels together. Adding these two nucleus-specific reporter signals resulted in a strong constitutive nuclear signal, allowing cells to be efficiently segmented and computationally tracked throughout the entire cell cycle.

**Figure 5:**
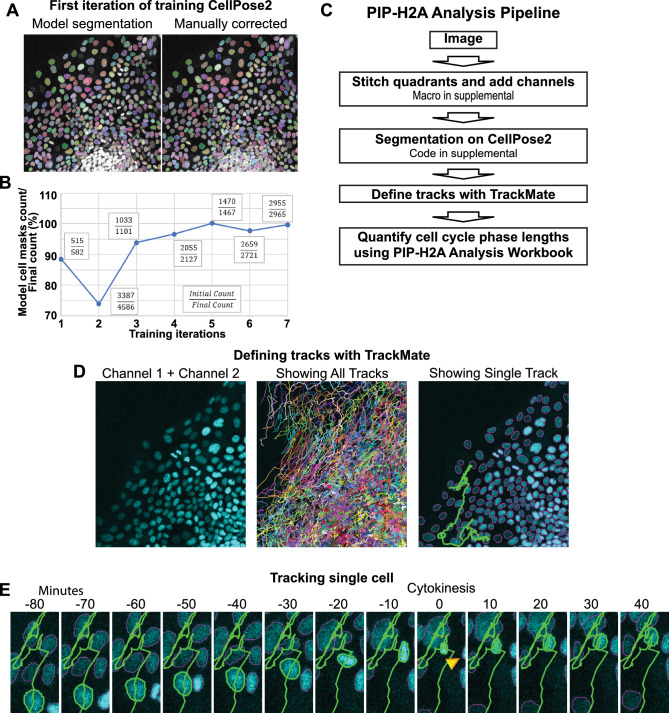
Analysis pipeline for PIP-H2A live imaging. (**A**) (Left) Example image from untrained CellPose2 model segmenting nuclei in human IEC monolayers. Segmented nuclei are highlighted with a colored mask. (Right) Final segmentation following human correction. (**B**) Readouts of mask numbers obtained by CellPose2 segmentation modeling/human-corrected final segmentation across 7 training iterations. (**C**) Schematic of the PIP-H2A live imaging analysis pipeline. (**D**) Example results from Trackmate showing nuclei defined by adding the mVenus and mScarlet channels (left), all defined tracks superimposed onto the nuclei (middle), then a single superimposed track for one nucleus (right). (**E**) Timelapse imaging showing one nucleus following its defined track across two hours through a cytokinesis event between time − 10 min and 0 min. Yellow arrowhead annotates the sister cell from the cytokinesis.

The CellPose2 software package^25^ was used to segment nuclei on individual frames of our IEC monolayer live imaging results. Human-in-the-loop training was utilized, where CellPose2 segments a new image, a researcher manually corrects errors, and the model learns from these corrections to improve segmentation in future iterations. Example images of the initial nuclei segmentation and manual corrections performed at iteration 1 are shown in Fig. 5A. To track training progression, the initial count of nuclei segmented by the model and the final corrected count were recorded for each iteration (Fig. 5B). In the 5th-7th training iterations, the model consistently reached > 95% accuracy and was considered adequately trained. Once the model was trained using single images, we developed code to analyze all frames of the live imaging file consecutively (Supplemental File 5), resulting in a TIF file with nucleus masks defined across all timepoints of the live imaging run.

The next step in our pipeline (Fig. 5C) was to analyze reporter signals across time for each nucleus. We used the TrackMate Fiji plugin^26,27^. This program uses the segmentation channel to track individual nuclei across frames based on object proximity between frames. To select tracks likely to follow a single nucleus through a full cell cycle, no gaps were allowed between frames and all tracks under 480 min were removed from analysis, since no cell cycle lengths below 500 min were found across all previously analyzed PIP-FUCCI IECs. Tracks under 480 min largely only covered a portion of a cell cycle before being lost due to improper segmentation by CellPose2 or by TrackMate failing to correctly assign the track due to the close proximity and dynamic motion of primary human IEC monolayers, especially around cytokinesis events. To ascertain that all tracks included in our final analysis were of a single nucleus that completed a full cell cycle, all were manually viewed. Our live imaging results showed that the mScarlet-H2A reporter experiences an acute spike in mean intensity as chromatids condense and split in mitosis, allowing mitotic events and total cell cycle length to be readily tracked from the mScarlet channel alone (Fig. 4E). Using this pipeline, we analyzed 284 high-quality tracks, showing that freely cycling IECs had a median total cell cycle length of 17.2 h, with first and third quartile lengths of [15.0, 20.4]. Median individual cell cycle phase lengths were 8.3 h [6.2,11.3] for G1 phase, 6.0 h [5.5, 6.5] for S phase, and 2.5 h [2.2, 3.0] for G2/M phases (Fig. 4F). A schematic of the full pipeline for analyzing PIP-H2A live imaging data is shown in Fig. 5C, and sample files are available as Supplementary Files 5–7.

### Mitochondrial respiration affects cell cycle length in human IECs

**Figure 6:**
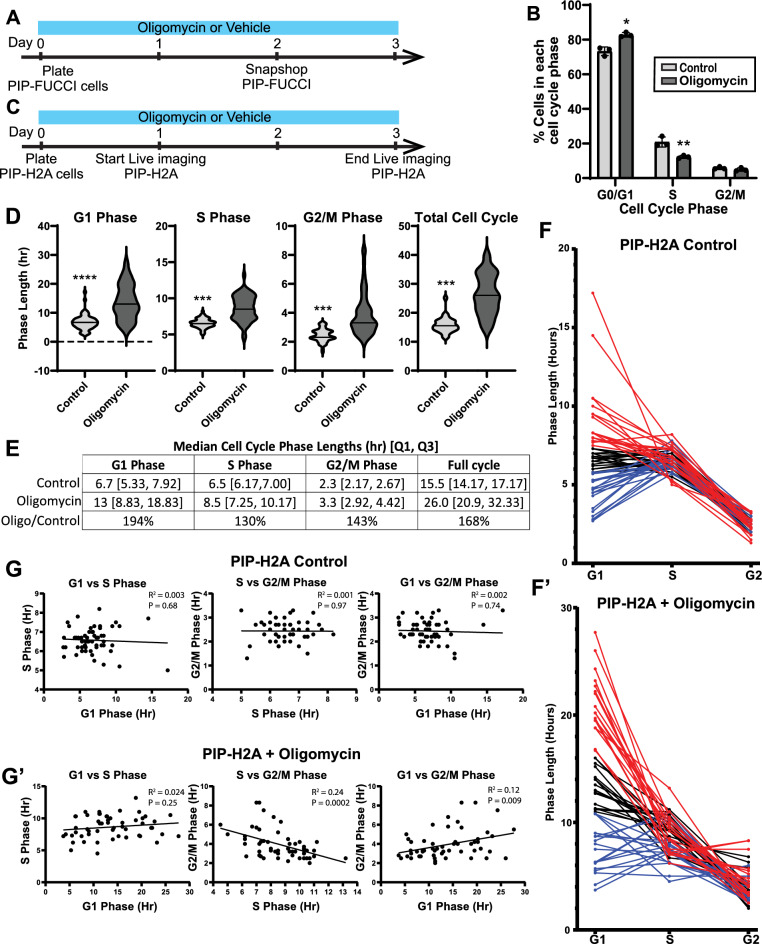
Inhibiting mitochondrial respiration slows cell cycle in IECs. (**A**) Timeline for PIPFUCCI cell treatment and imaging. (**B**) Ratios of cells in each cell cycle phase as obtained from a snapshot of PIPFUCCI IECs following two days of oligomycin treatment. (**C**) Timeline for PIP-H2A cell treatment and imaging. (**D**) Violin plots denoting cell cycle phase lengths across 55 IECs from N = 1 well per condition using the PIP-H2A reporter. (**E**) Median and [1st quartile, 3rd quartile] cell cycle phase lengths in control or oligomycin-treated IECs and ratios of oligomycin-treated vs control lengths. F) Phase lengths for individual control (**F**) and oligomycin-treated (**F**’) cells with lines connecting cycle lengths for each cell. Red = 1/3 cells with the longest G1 phase, Blue = 1/3 cells with the shortest G1 phase. (**G**) Scatter plots with line of best fit shown comparing each cell cycle phase length for all control (**G**) and oligomycin-treated (**G**’) cells. Linear regression was performed and R^2^ and *P* values are shown for each comparison.

Mitochondrial respiration is increasingly shown to regulate proliferation and stemness in IECs^28–30^. To quantify how inhibiting mitochondrial respiration affects cell cycle in primary human IECs, we treated IECs with oligomycin, a common ATP synthase inhibitor. PIP-FUCCI reporter IECs were plated into chamber slides and immediately treated with oligomycin or vehicle control. Importantly, we utilized our multi-well chamber slide format to grow control and treated IECs side-by-side to avoid differences in culture conditions or plating density. To utilize the ability of the PIP-FUCCI construct to report cell cycle in a snapshot of living cells, growing IECs were imaged at 48 h post-plating (Fig. 6A,B). Image analysis of control IECs showed 73.3 ± 2.5% of nuclei were mVenus^+^ (G1/G0 phases), 20.9 ± 2.9% were mCherry^+^ (S phase), and 5.8 ± 0.7% were mVenus^+^mCherry^+^ (G2/M phases). In contrast, oligomycin-treated IECs showed 82.8 ± 1.3% mVenus^+^ nuclei, 12.3 ± 0.8% mCherry^+^, and 4.9 ± 1.0% mVenus^+^mCherry^+^ (Fig. 6C). The 9.5% increase in G1/G0 cells and 8.5% decrease in S phase cells demonstrates that oligomycin leads to an overall shift in the population from S to G1 phase, but it is insufficient to determine whether this is due to cell cycle arrest in G1 or phase elongation, and changes to all cell cycle phase lengths cannot be determined due to the large change in the G1/G0 population.

To gain a better understanding of how oligomycin affects cell cycle, we live-imaged PIP-H2A IECs from 24 to 76 h post plating with oligomycin or vehicle. Our pipeline allowed us to directly quantify cell cycle phase lengths across growing IECs. We found that oligomycin significantly increased the length of all phases of the cell cycle in cells that completed a full cell cycle within the analysis window (Fig. 6D,E). Under oligomycin treatment, the median G1 phase lengthened 94%, from 6.7 h in control IECs to 13 h in oligomycin-treated IECs; the median S phase lengthened 31%, from 2.3 to 3.3 h; the median G2/M phases lengthened 43%, from 2.3 to 3.3 h; and the median total cell cycle length increased from 15.5 to 26.0 h. Thus, using our PIP-H2A reporter pipeline to quantify all phase lengths demonstrated that the increased G1/G0 proportion indicated by the PIP-FUCCI cells results from actively cycling cells spending a longer time in G1 phase as opposed to oligomycin solely arresting cells in G1, and lesser increases to S and G2/M phase lengths also became apparent.

The wider spread between phase lengths across IECs treated with oligomycin prompted us to analyze whether lengths of all cell cycles correlate within individual cells. To visualize whether there were obvious patterns, we graphed phase lengths for each cell and connected the cell cycle phases for each individual cell, with the 1/3 cells with the longest G1 phases colored red and the 1/3 with shortest G1 colored blue (Fig. 6F). No obvious patterns were observed, so we next compared each cell cycle phase across cells using scatter plots with linear regression analyses (Fig. 6G). No correlations were seen between cell cycle phase lengths in control cells, with all linear regression lines statistically similar to zero. Interestingly, oligomycin treatment skewed these results. Cells with longer S phases were shown to have shorter G2/M phases, and those with longer G1 phases correlated with longer G2/M phases.

Differences in cell cycle length were observed between experiments using reporter IECs (Table 1). Changes to total cell cycle readouts and those for individual phase lengths were observed between seperate assays using the same PIP-H2A clonal cells as well as between PIP-H2A and PIP-FUCCI platforms. This exemplifies the importance of using a platform that allows for internal controls for each experiment to allow for testing control and experimental cells on the same slide to avoid differences in collagen, media, plating density, or other perturbations.

**Table 1:** Comparing median cell cycle phase lengths between experiments.

	Median cell cycle phase lengths (hr) [Q1, Q3]			
	G1 phase	S phase	G2/M phase	Full cycle
PIPFUCCI (Fig. 3)	7.7 [5.8, 10.3]^a^	8.2 [7.2, 8.8]^c^	2.7 [2.2, 3.3]^e^	18.5 [16.5, 21.5]^g^
PIP-H2A (Fig. 4)	8.3 [6.2, 11.3]^a,b^	6.0 [5.5, 6.5]^c,d^	2.5 [2.2, 3.0]^e,f^	17.2 [15.0, 20.4]^g,h^
PIP-H2A (Fig. 6)	6.7 [5.3, 7.9]^b^	6.5 [6.2,7.0]^d^	2.3 [2.2, 2.7]^f^	15.5 [14.2, 17.2]^h^

Median and [1st quartile, 3rd quartile] cell cycle phase and total cycle lengths from each experiment in the paper. Significance was tested between PIP-H2A and PIP-FUCCI and between both PIP-H2A experiments. Paired superscript letters denote the two values are significantly different with *P* < 0.05.

## Discussion

In this study, we transcriptionally interrogated collagen-plated human IEC cultures, genetically engineered primary human IECs with two cell cycle reporter constructs, optimized a microphysiological platform for live imaging these cells, then compiled a pipeline using freely available programs to measure cell cycle phase lengths. The ability to measure cell cycle phases in freely cycling primary human IECs represents an important advance, as IEC cell cycles have only been visualized using model organisms in current literature. Our human cell cycle data closely resembles data from intestinal organoids derived from FUCCI2 mice^17^. While this murine study did not report individual phase lengths, they report an average total cell cycle length of 18.4 h in murine organoids. This total length is very similar to those defined by our human platform. They also report a large portion of cells cycle around 15 h with another group taking slightly over 20 h, mirroring our data from both PIP-FUCCI (Fig. 3F) and PIP-H2A (Fig. 4F) human IECs. This highlights similarities between mouse and human IECs and demonstrates the strength of concurrently tracking cell cycle phases across individual IECs.

scRNAseq results from primary IECs cultured as monolayers on soft collagen indicate that these cultures maintain a broad population of stem and progenitor cells (Fig. 2). While many genes were originally demonstrated to mark ISCs in mice, our recent scRNAseq atlas supports that these markers are conserved in primary human ISCs^23^. IEC monolayers containing stem cells defined by gene markers is consistent with our earlier study demonstrating that IECs plated as monolayers retain functional stem cell activity, with a 2–3% organoid formation efficiency^31^. The presence of both stem and progenitor cells in our IECs might also explain the biphasic cycle lengths shown by both reporters (Figs. 3F and 4F), with the population cycling > 20 h possibly representing ISCs and the population < 20 h possibly representing TA-like cells. Our reporters open opportunities to probe these populations further in future work.

Analysis pipelines developed for other FUCCI models are largely inadequate to account for the specific needs of imaging tightly packed and highly motile primary IECs. For example, one study reports an elegant TrackMate strategy similar to ours to analyze several FUCCI permutations in largely immobile HeLa cells and mouse embryonic stem cells^13^. Our pipeline necessitated several modifications. Capturing the motion of freely migrating IEC monolayers across 48 h required a much wider imaging area, so we used a 20 × objective instead of the 40 × used in their study. The lower resolution imaging resulted in the need to use CellPose2 to segment the images before TrackMate analysis and also raised the issue of not being able to track moving nuclei through a fluorescence gap in G1 phase, as discussed above. Another main difference is that our pipeline does not allow any frame gaps in TrackMate to minimize occurrences where the tracked segment skips from one cell to another cell as IECs migrate, and we further follow TrackMate by manually viewing each positive track to prune such events.

Our PIP-FUCCI and PIP-H2A constructs present opportunities for diverse applications. The PIP-FUCCI construct is optimal for snapshot analyses where proportions of the IEC population in each cell cycle phase can be determined from a single image. Live cells can be imaged at different timepoints, such as before and after a drug is added, gene function is induced, or injury occurs. This genetic construct could also easily be scaled up into plates with more wells for drug screens or other large assays, with snapshots across wells analyzed to determine effects on cell cycle phases in primary human IECs. Also, unlike DNA content analysis, which relies on population-level calculations to define which cells reside in each phase^32^, our live reporter directly indicates the current phase for each individual cell. If direct measurements of cell cycle phase lengths are needed, the PIP-H2A construct is better suited for live image analysis than the PIP-FUCCI construct. Measuring cell cycle phase lengths in individual cells can give more detailed information than ratiometric snapshot or DNA content analysis. To expand future applications, we avoided simply adding a far-red constitutive reporter to the original PIP-FUCCI cells to leave an open fluorescent channel for combining with fluorescent stains or additional reporters.

The oligomycin treatment highlighted many strengths of our platforms. PIP-FUCCI IECs showed cell cycle differences without need for fixing, staining, or FACS (Fig. 6B). The PIP-H2A platform provided additional detail, showing that all phases lengthened upon oligomycin treatment (Fig. 5D,E). This result is impossible to observe from ratiometric DNA content analysis alone. The PIP-H2A platform also only measures actively cycling cells, while DNA content analysis or PIP-FUCCI snapshot quantifications include cells which have exited the cell cycle. Quantifying across individual cells also allows for population dynamics to be visualized. We saw increased phase length heterogeneity upon oligomycin treatment, and closer analysis showed that a subpopulation of cells with a larger increase in S phase length experienced statistically shorter G2/M lengths (Fig. 6D–G). This indicates that oligomycin is having different effects on subpopulations within our IECs, as no novel correlations would arise if oligomycin equally increased the cycling time of all IECs. Our platform could be used to further probe subpopulations by pairing our two-color reporters with additional dyes or reporters for mitochondrial content, ISC vs TA identity, or other properties to elucidate the cause of these differences.

While our new pipeline has substantially increased throughput compared to manual tracking, there are some limitations. The main difficulty is the movement and proximity of nuclei in the primary human IEC monolayers. Cells migrate quickly, and cytokinesis events occur within the plane of the monolayer, resulting in dynamic movement as separating cells jostle neighboring cells. For these reasons, we watched each analyzed track by eye to ascertain that a single nucleus was followed for a full cell cycle. The dynamics of the H2A-mScarlet signal readout made this easier, as we could quickly scan for tracks that have the tight peaks stereotypical of chromatid formation and separation to choose tracks most likely to track a full cell cycle (Fig. 4E). Tracks can also be lost as a result of our stringent filtering rules prohibiting any gaps in tracking. We expect these caveats to be overcome as segmentation and tracking technologies improve, and future studies can also use a shorter imaging time (< 10 min/frame) to minimize the distances nuclei move between frames.

This study presents a full platform for analyzing cell cycle phase lengths in freely cycling primary human IECs. We provide files for 3D printing collagen presses and for our analysis pipeline to allow other labs to use this technique. Accurately delineating cell cycle phase lengths is important for studying the intestinal epithelium in health and disease, identifying cell cycle regulators in IECs, and defining the effects of drugs and other molecules on cell cycle.

## Methods and materials

### Tissue procurement, dissection, and crypt isolation

Human intestinal crypts were harvested following a published protocol^20^. Donor-grade human intestines were received from HonorBridge. The proximal 9 cm of the small intestine was designated duodenum, with jejunum defined as the upper half of the remaining small intestine. A 3 × 3 cm piece was resected from the middle of the jejunum then stored in Advanced DMEM/F12 [Gibco 12634-010] + 10 µM Y27632 [Selleck Chemical S6390] and 200 µg/mL Primocin [Invivogen ant-pm-05] on ice. The tissue was incubated in PBS [Gibco 14190-144] + 10 mM *N*-acetylcysteine [Sigma-Aldrich A9165] for 15 min then transferred to Isolation Buffer (5.6 mM Na_2_HPO_4_ [Sigma S7907], 8.0 mM KH2PO4 [Sigma P5655], 96.2 mM NaCl [Sigma S5886], 1.6 mM KCl [Sigma P5405], 43.4 mM Sucrose [Fisher BP 220-1], and 54.9 mM d-sorbitol [Fisher BP 439-500]) + 2 mM EDTA [Corning 46-034-Cl] + 0.5 mM DTT [Fisher Scientific BP 172-5] for 30 min with gentle rocking at room temperature followed by vigorous shaking for 2 min. After shaking, the tissue was transferred to a new tube of Isolation Buffer + EDTA + DTT and the rocking then shaking were repeated six times. Supernatants for each were stored on ice. Supernatants were checked via light microscope for the presence of crypts and villi. Crypt-enriched shakes were pooled, washed in Isolation Buffer, then cultured or frozen.

### Tissue culture

Primary human jejunal stem cells from a 34 year old male with no known gastrointestinal diseases were cultured in collagen coated well plates prepared following the protocol in^18^. Maintenance media was prepared as published^33^. Briefly, L cells expressing transgenic Wnt3A, Noggin, and R-spondin3 (ATCC CRL-3276) were cultured in Collection Media (20% Tetracycline-negative Fetal Bovine Serum [Gemini 100-800], 1% Glutamax [ThermoFisher 35050061], 1%Pen/Strep [ThermoFisher 15070063], in Adv DMEM/F12) for 12 days, with media collected daily. Maintenance Media (MM) consisted of 50% conditioned Collection Media and a final concentration of 2% B-27 Supplement [ThermoFisher 12587001], 5 mM Nicotinamide [Sigma-Aldrich N0636], 10 mM HEPES [Corning 25-060-CI], 1 mM Glutamax, 1X Pen/Strep, 0.6125 mM *N*-Acetylcysteine, 25 µg/mL Primocin, 1.5 µM, 25 ng/mL mEGF [Peprotech 315-09], 1 nM Gastrin [Sigma-Aldrich G9145], and 5 nM Prostoglandin E2 [Peprotech 3632464]. Fresh crypts were plated with 200 mg/mL Primocin, 200 mg/mL Gentamycin [Sigma-Aldrich G1914], and 0.5 mg/mL Amphotericin B [Sigma-Aldrich A2942] for the first week. Cells were grown in a humidified incubator at 37 °C with 5% CO_2_. Cells were passaged every 4–6 days in a 1:3 or 1:4 ratio using Collagenase IV [ThermoFisher LS004189] to digest the collagen patty, dPBS to wash the cells, TrypLE [Gibco 12605-010] and trituration with a P1000 pipet to dissociate cells, then plating into new fresh collagen wells. Cells were frozen following the normal dissociation steps but then resuspending cells in 10% DMSO [Sigma D2650], 30% FBS, 60% MM + 1:1000 Y27632. Cells were frozen in a -80 C Freezer overnight encased in Styrofoam then stored in liquid nitrogen. Cryovials were thawed in 37 C water until most of the ice melted then the contents immediately transferred to 8 ml MM, pelleted, then plated in MM + Y27632. Cells from the parent line at equivalent passages used in these studies were verified to maintain the ability to differentiate into all expected cell types when cultured in differentiation media.

### Transcriptomics

Cells analyzed in this experiment were previously published as part of the dataset in Gomez et al^22^ and available on the NCBI Gene Expression Omnibus with GEO Accession GSE186583. The published dataset included multiple distinct samples of cells that were sequenced in one run, distinguishable by Hashtag oligo-antibody staining, as previously performed^23^. The transcriptomic readouts from Gomez et al. were subsetted down to just IECs grown on soft collagen with no differentiation media (i.e. ‘hash_id’ = = 1) to view general proliferative and stem cell traits. The dimensional reduction from Gomez et al. was preserved and overlaid with genes of interest, and annotations for cell-cycle phase were determined using previously published methods^34–36^. To produce this dataset, wildtype jejunal human IECs were grown on soft collagen in a 6w plate until 70% confluent. To dissociate to single cells, collagen was digested with collagenase for 20 min as above, washed, then dissociated in 1 mL of 4 mg/mL cold protease in DPBS with vigorous pipetting every 2 min until 80 + % single cells were observed via light microscope. After dissociation, cold protease was quenched with Advanced DMEM/F12 + 1% FBS. After washing, cells were FACS sorted using forward scatter and side scatter to gate for single cells and Annexin V to gate out dead cells. Transcriptomic data analysis was then performed using the Chromium Next GEM Single-Cell 3’ GEM, Library and Gel Bead Kit v3.1 (10X Genomics) following our previous pipeline^22,23^.

### Genetic engineering and cloning

Genetic engineering followed established protocols^20^. In short, DNA segments of importance were extracted using restriction enzymes or amplified utilizing CloneAmp HiFi PCR Premix [Takara 639298]. Plasmids were engineered using an In-Fusion HD Cloning Kit [Takara 638920] to align the reporter gene between inverted terminal repeats (ITR) to allow for transposing into the genome via super-piggyBac transposase. Plasmids were subsequently harvested from bacterial stocks using the QIAGEN HiSpeed Maxi kit [Qiagen 12662].

The PIP-FUCCI plasmid^11^ was a gift from Jean Cook and Jeremy Purvis. pPIGA-PHD was a gift from Linzhao Cheng [Addgene plasmid #26778; http://n2t.net/addgene:26778; RRID:Addgene_26778]^37^. sg resistant gamma-tubulin was a gift from Maria Alvarado-Kristensson [Addgene plasmid #104433; http://n2t.net/addgene:104433; RRID:Addgene_104433], and pmScarlet_H2A_C1 was a gift from Dorus Gadella [Addgene plasmid #85051; http://n2t.net/addgene:85051; RRID:Addgene_85051].

Plasmids were introduced into human cells using our optimized protocol^20^ employing the Neon Transfection System 100 μL Kit [ThermoFisher MPK10096]. Dissociated cells were suspended in 100 μL Neon Buffer R at 10,000 cells/μL with 6 μg of plasmid. Super PiggyBac Transposase Expression Vector [System Biosciences PB210PA-1] (5 ng/μL) was used for all transfections. Cells were electroporated using Neon preset #5 (1700 V, 1 pulse, 20 ms) then transferred to a collagen-coated plate containing MM + Y27632.

After 4–7 days following transfection, colonies were subjected to selection using Blasticidin (10 μg/mL) [Gibco A1113903] for 4–8 days. Surviving colonies were isolated by digesting the collagen with 100 μL/mL collagenase IV ( 5000 U/mL) at 37 °C for 25 min, washed with dPBS, then individual colonies were picked using a 20 μL pipette on a light microscope. Isolated colonies were placed into separate collagen-coated 48-well plate wells with 300 μL of MM + Y27632.

### Staining

Monolayers were pulsed with 10 µM EdU [Molecular Probes C10634] for 1 h, fixed for 20 min with 4% PFA [Thermo Scientific AC416780250] then washed and stored in dPBS at 4 °C until staining. Cells were permeabilized with 0.5% Triton X-100 [Fisher Scientific BP151-100] then stained for EdU Reaction Buffer (4 mM CuSO4 [Fisher Scientific S25286], 2 µM Sulfo-CY5-azide [Lumiprobe A3330], 0.2 M Ascorbic Acid [Fisher Scientific AC352681000], in PBS) for 1 h at room temperature protected from light, washed with PBS, stained with DAPI [Invitrogen D3571], then imaged on the Andor Dragonfly Spinning Disk Confocal Microscope. In multiple image panels, all were taken with the same microscope settings and had the same display adjustments made. Images were analyzed using FIJI software^38^.

### Collagen press

To reproducibly coat an Ibidi µ-Slide 4 Well chamber slide [Ibidi 80426] with 0.3 mm collagen, a custom collagen press was created and fabricated using chlorinated polyethylene and an Ultimaker S3 3D printer (Supplemental File 1). The feet of the press were affixed with coverslip glass using Glass Glue [Loctite], and Rain-X [Rain-X 800002250] was applied following the manufacturer's guidelines to allow for removing the press without pulling up the thin collagen layer. Collagen was prepared according to a published protocol^18^. Each chamber received 250 μL of liquid collagen, the press was immediately positioned on top (as in Fig. 1), then the chamber slide with the press was moved to a 37 °C incubator for 90 min for the collagen to solidify. Afterward, the presses were gently detached and dPBS was added to cover the collagen. Prepared slides were stored at room temperature in zip-top bags until use.

### Live imaging

IECs transfected with PIP-H2A fluorescent reporters were seeded onto collagen-coated chamber slides 24–48 h before imaging, and growth media was changed the morning of the imaging session. Live imaging was performed on an Andor Dragonfly Spinning Disk Confocal Microscope mounted on a Leica DMi8 microscope stand, using a Leica HC PL APO 20x/0.75 LWD air objective with pinhole size set to 40 μm. The camera was an Andor iXon Life 888 EM-CCD, with Electron Multiplying Gain set to 150, Horizontal Shift Speed of 10 MHz–16 bit, 2X Pre Amp Gain, 2.2 μs Vertical Shift Speed, Normal Vertical Clock Voltage and binned 2 × 2. Live imaging was begun on fairly small colonies to allow room for growth and minimizing contact inhibition during the imaging timeframe.

To image the mCherry gene of the PIP-FUCCI construct and the mScarlet gene in the PIP-H2A construct, a 561 nm laser was used for excitation, and light was collected with a 593/43 Semrock emission filter using a HC Fluotar L 25X/0.95 W 0.17 VISIR objective. For mVenus imaging, a 514 nm laser was used for excitation, and light was collected with a 538/20 Semrock emission filter. Images had 512 × 512 pixels and 1.00 μm pixel size. At each position, Z stacks were acquired using a piezo Z stage with 5 μm intervals spanning 75 μm (16 steps). 2 × 2 montages were acquired at each position. Each Z stack in the 2 × 2 montage overlapped its neighboring Z stacks by 10%. Two locations in each well were acquired per experiment. 2 × 2 montage Z stacks at each location were acquired every 10 min for 48 h. All images directly compared to each other were acquired using the same settings. Typical settings were 600 ms exposures with 5% laser power for mScarlet, and 200 ms exposures with 1% laser power for mVenus. Temperature was maintained at 37 °C with an Okolab microscope enclosure, with continuous monitoring and feedback. 5% CO2 was warmed to 37 °C in the enclosure and humidified before being delivered to an enclosed stage top holder that contained the sample. To minimize evaporation during the experiment, 8 caps from 15 mL falcon tubes were filled with water and placed surrounding the sample inside the stage-top sample holder.

### Live-imaging analysis

*PIP-FUCCI construct:* Stitched Z stacks were analyzed using Bitplane Imaris software (version 9.9.1) and Microsoft Excel. A full analysis protocol, a custom Excel analysis workbook, and a sample analyzed Imaris file are included as Supplemental Files 2–4. Z slices without nuclear fluorescent signal were cropped out, and remaining Z stacks were max projected along the XY plane. Individual nuclei at each timepoint were marked manually across timepoints using the “spots” function in Imaris, then tracks were generated to connect the manually marked nuclei throughout the experiment. Mean fluorescent intensities were exported from Imaris into a custom Excel workbook to determine cell cycle phases. Cells were only tracked from mother cells which divided at least 5 h after the start of imaging to allow cells to acclimate to the imaging environment. Tracking was also only started on mother cell divisions that occurred at least 20 h prior to the end of the video. Cells were chosen from a variety of spots across the viewing area and timelapse for tracking. An average of 90 nuclei were tracked at each location per experiment (range = 59–106). The Excel workbook presents graphs of normalized mVenus and mCherry fluorescence. The G1-S transition was defined as the point after mVenus signal dropped over 50%. The S-G2 transition was defined as the point when the mVenus signal began rising above the lowest maintained level again. Final graphs were made using GraphPad Prism 9. Data is shown as violin plots for each cell cycle phase and for total cell cycle duration, with median values shown.

*PIP-H2A construct*: Images were max projected for maximum intensity then stitched using FIJI software, with 10% overlap between quadrants. To maximize nuclear signal for tracking, the mScarlet and mVenus channel intensities were added together using FIJI to form a new channel. This summed and stitched channel was imported to CellPose2 for segmentation. An automated Macro to run the previous steps on FIJI is available in Supplemental Fig. 5. To train the CellPose2 model, seven individual frames were used including individual and stitched positions. The “nuclei” option from the model zoo was chosen, with average nuclei diameter set to ‘12’. For training, the model predicted nuclei segmentation then a researcher would correct the results. Initial and final nuclei counts were recorded for each training session. Upon reaching > 95% accuracy for three consecutive rounds, the model was considered trained. A Jupyter Notebook script was used to run the CellPose2 analysis on all 488 frames of the time-lapse image individually and save the resulting masks as a single TIFF file with 488 frames (Supplemental File 5). Nuclei were tracked across frames using the Fiji Trackmate Plugin. To begin, a four channel TIFF was made combining C1: mScarlet, C2: mVenus, C3: C1 + C2, C4: segmentation from Trackmate across timepoints, then imported into Trackmate. No cropping was chosen, and the Label Image Detector function was chosen using the segmentation masks in channel 4 to define nuclei. No initial thresholding or filtering was done prior to defining tracks. The ”LAP Tracker” was chosen, with specifications set to 10um max distance, Gap close = 7.0, and 0 gaps allowed. After tracks were defined, tracks with total duration < 480 min were filtered out as that was shorter than the shortest cell cycle recorded using the PIP-FUCCI reporter. To choose tracks of nuclei which underwent a full cell cycle within the viewing period, Track name, frame, and Max Intensity of C1 values were all exported to Microsoft Excel then filtered for tracks which had at least one point where slope >|300|, following observations of dynamically brighter mScarlet signal during cytokinesis (Fig. 3E). Candidate tracks were monitored by eye to ascertain that the track followed a single nucleus through two splitting events, then raw data for the track was copied to an Analysis Workbook in Microsoft Excel which presents graphs of normalized mVenus and mScarlet fluorescence (Supplemental File 6 and 7). The G1-S transition was defined as the point after mVenus signal dropped over 50%. The S-G2 transition was defined as the point when the mVenus signal began rising above the lowest maintained level again. Cytokinesis was defined by acute peaks in mScarlet intensity. Data full imaging run for PIP-H2A including stitched and max-projected time lapses for mVenus and mCherry data, channel 3 resulting from adding both signals together, and the results of CellPose2 segmentation over the full timelapse, as well as the file resulting from TrackMate analysis of the timelapse imaging to so nucleus tracking, are available on Zenodo at 10.5281/zenodo.11506667

### Oligomycin treatment

Cells were treated with 2.5 μM oligomycin [Sigma O4876-5MG] or an equivalent amount of vehicle (dPBS) as they were sparsely plated in chamber slides. Media and treatments were replaced the following morning. For PIP-FUCCI analysis, cells were imaged 48 h post plating. All nuclei in three images per condition (using a 25 × objective, 350 + nuclei/image) were visually determined whether each nucleus had coloration above the background noise for mVenus, mCherry, or both, then ratios calculated using Excel. For PIP-H2A analysis, cells were live imaged from 24 h through 72 h post plating, with oligomycin present throughout. 55 nuclei that completed a full cycle within the imaging period were quantified for each condition, following the power analysis below.

### Statistics

The absence of direct prior research comparing cell cycle phase lengths in IECs treated with oligomycin necessitated an estimation of the effect size, which was calculated to be 0.714 based on experimental data comparing wild-type human intestinal cells with SOX9 knockout^39^ where the two groups exhibited mean cell cycle lengths of 8.436 and 5.796 h, respectively with a standard deviation of approximately 3.7 within each group. To achieve robust and reliable results, the power analysis aimed for a significance level (α) of 0.05 and a power of 0.95, which is stringent enough to detect even modest effects of the drug treatment. A priori analysis was conducted using G*Power version 3.1.9.7^40^ to calculate a minimum sample size of 55 per group. This sample size ensures a high probability of correctly rejecting the null hypothesis, should a true effect exist, thereby minimizing the risk of Type II errors.

To compare cell cycle phase lengths, normality was first tested using the Shapiro Wilk test for each cell cycle phase of each population. Comparisons in which both populations showed normality were analyzed using a student’s t test. Comparisons with at least one group not showing normality were analyzed using a two-tailed Wilcoxon–Mann–Whitney. *indicates *P* < 0.05, **indicates *P* < 0.005, ***indicates *P* < 0.0005. Oth

To analyze correlations between cell cycle phase lengths across individual cells, a simple linear regression was performed on scatter plots comparing lengths of two cell cycle phases using GraphPad Prism 9.3.1. A *P* value < 0.05 for the slope being significantly non-zero denotes a statistically significant correlation between two phases.

Final graphs and significance analyses were made using GraphPad Prism 9. Data is shown as violin plots for each cell cycle phase and for total cell cycle, with median values shown. Median values and quartile ranges provided were calculated using Excel.

### Supplementary Information

Supplementary Legends.

Supplementary Information 1.

Supplementary Information 2.

Supplementary Information 3.

Supplementary Information 4.

Supplementary Information 5.

Supplementary Information 6.

Supplementary Information 7.

## Data Availability

The corresponding authors can be reached with requests for materials at joseph.burclaff@med.unc.edu and scott_magness@med.unc.edu. Materials and resources made for this project will be made available upon reasonable request. All code used for this project is available in Supplemental File 5. Example data for tracking cell cycle are available on Zenodo at 10.5281/zenodo.11506667
