# Supplementary material for: An in vitro platform for quantifying cell cycle phase lengths in primary human intestinal epithelial cells
Source: Sci Rep. 2024 Jul 2;14:15195. doi: 10.1038/s41598-024-66042-9 (PMC11219882; doi:10.1038/s41598-024-66042-9)
Supplement: Supplementary file 1 — Supplementary Legends. [file 41598_2024_66042_MOESM1_ESM.docx]

**Supplemental File 1: Collagen press design file**

**Supplemental File 2: PIP-FUCCI analysis protocol**

**Supplemental File 3: PIP-FUCCI empty analysis workbook**

**Supplemental File 4: Example PIP-FUCCI analysis workbook**

**Supplemental File 5: Code files for PIP-H2A live imaging analysis**

**Supplemental File 6: PIP-H2A empty analysis workbook**

**Supplemental File 7: Example PIP-H2A analysis workbook**
